# Early‐onset coenzyme Q10 deficiency associated with ataxia and respiratory chain dysfunction due to novel pathogenic *COQ8A* variants, including a large intragenic deletion

**DOI:** 10.1002/jmd2.12107

**Published:** 2020-06-02

**Authors:** Ana Cotta, Charlotte L. Alston, Sidney Baptista‐Junior, Julia F. Paim, Elmano Carvalho, Monica M. Navarro, Marie Appleton, Yi Shiau Ng, Jaquelin Valicek, Antonio L. da‐Cunha‐Junior, Maria I. Lima, Alessandra de la Rocque Ferreira, Reinaldo I. Takata, Iain P. Hargreaves, Gráinne S. Gorman, Robert McFarland, Germaine Pierre, Robert W. Taylor

**Affiliations:** ^1^ Department of Pathology SARAH Network of Rehabilitation Hospitals Belo Horizonte Brazil; ^2^ Wellcome Centre for Mitochondrial Research Translational and Clinical Research Institute, Faculty of Medical Sciences, Newcastle University Newcastle upon Tyne UK; ^3^ NHS Highly Specialised Services for Rare Mitochondrial Disorders, Royal Victoria Infirmary Newcastle upon Tyne Hospitals NHS Foundation Trust Newcastle upon Tyne UK; ^4^ Department of Neurophysiology SARAH Network of Rehabilitation Hospitals Belo Horizonte Brazil; ^5^ Department of Pediatrics and Genetics SARAH Network of Rehabilitation Hospitals Belo Horizonte Brazil; ^6^ Clinical Biochemistry, Royal Victoria Infirmary, Newcastle upon Tyne Hospitals NHS Foundation Trust Newcastle upon Tyne UK; ^7^ Department of Radiology SARAH Network of Rehabilitation Hospitals Belo Horizonte Brazil; ^8^ Department of Electron Microscopy SARAH Network of Rehabilitation Hospitals Brasília Brazil; ^9^ Department of Molecular Biology SARAH Network of Rehabilitation Hospitals Brasília Brazil; ^10^ Neurometabolic Unit National Hospital for Neurology and Neurosurgery London UK; ^11^ School of Pharmacy and Biomolecular Sciences, Liverpool John Moores University Liverpool UK; ^12^ South West Regional Metabolic Department Bristol Royal Hospital for Children Bristol UK

**Keywords:** ataxia, CoQ10, *COQ8A* deletion, encephalomyopathy, mitochondrial disease

## Abstract

Coenzyme Q10 (CoQ10) deficiency is a clinically and genetically heterogeneous subtype of mitochondrial disease. We report two girls with ataxia and mitochondrial respiratory chain deficiency who were shown to have primary CoQ10 deficiency. Muscle histochemistry displayed signs of mitochondrial dysfunction—ragged red fibers, mitochondrial paracrystalline inclusions, and lipid deposits while biochemical analyses revealed complex II+III respiratory chain deficiencies. MRI brain demonstrated cerebral and cerebellar atrophy. Targeted molecular analysis identified a homozygous c.1015G>A, p.(Ala339Thr) *COQ8A* variant in subject 1, while subject 2 was found to harbor a single heterozygous c.1029_1030delinsCA variant predicting a p.Gln343_Val344delinsHisMet amino acid substitution. Subsequent investigations identified a large‐scale *COQ8A* deletion *in trans* to the c.1029_1030delinsCA allele. A skin biopsy facilitated cDNA studies that confirmed exon skipping in the fibroblast derived *COQ8A* mRNA transcript. This report expands the molecular genetic spectrum associated with *COQ8A*‐related mitochondrial disease and highlights the importance of thorough investigation of candidate pathogenic variants to establish phase. Rapid diagnosis is of the utmost importance as patients may benefit from therapeutic CoQ10 supplementation.

**SYNOPSIS**

*COQ8A* mutations cause pediatric ataxia with CoQ10 deficiency; carrier testing is vital to confirm recessive inheritance for accurate counseling.

## 1 INTRODUCTION

Primary coenzyme Q10 (CoQ10, ubiquinone) deficiency is a clinically and genetically heterogeneous subtype of mitochondrial disease with a variable age of onset.^1^ CoQ10 is important for many processes including oxidative phosphorylation, reactive oxygen species (ROS) scavenging and pyrimidine synthesis.^2^ Cerebellar ataxia is the most common clinical presentation associated with primary CoQ10 deficiency, but other clinical features include cardiomyopathy, encephalomyopathy, isolated myopathy and nephrotic syndrome.^1^, ^3^ While CoQ10 deficiency can occur secondary to statin therapy^4^ or alternative gene defects,^5^ most cases are caused by biallelic pathogenic variants affecting one of the nine enzymes involved in its biosynthesis via the mevalonate pathway.^6^, ^7^ Patients diagnosed with primary CoQ10 deficiency often benefit from therapeutic CoQ10 supplementation to ameliorate their symptoms.^8^, ^9^ Rapid diagnosis is important to ensure therapeutic interventions are implemented where possible. We report the biochemical, histochemical and molecular genetic findings relating to two young, female patients, both of whom presented with early onset cerebellar ataxia and were found to harbor recessive pathogenic variants in *COQ8A*.

## 2 MATERIALS AND METHODS

### 2.1 Case reports

**Figure 1:**
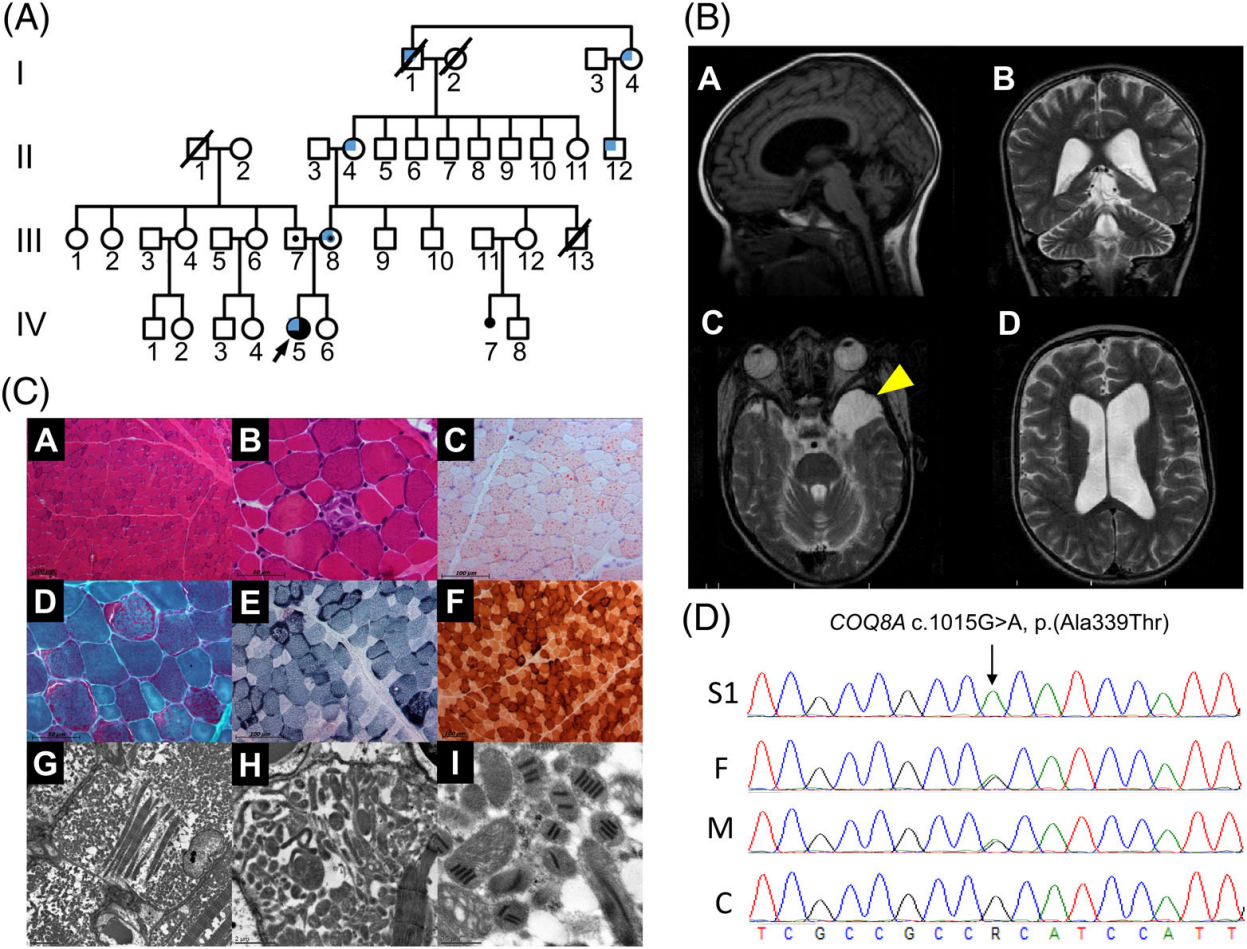
Pedigree, muscle histochemistry, neuroimaging, and molecular genetic findings for subject 1. A, Family pedigree for subject 1 reveals no evidence of consanguinity or additional affected individuals. Blue quadrant shading denotes the individuals reported to have glaucoma. B, Sagittal T1 weighted (panel A), coronal T2‐weighted (panel B) and axial T2‐weighted (panels C and D) MR brain imaging shows cerebellar atrophy, supratentorial ventriculomegaly, and a left temporal fossa arachnoid cyst (arrow head). C, Histochemical analysis of muscle biopsy shows preserved general architecture using H&E stain at 100× magnification (A), focal necrotic fibers using H&E stain at 400× magnification (B), pronounced lipid deposition on Oil‐red‐O staining at 200× magnification (C), ragged red fibers using Gomori modified trichrome staining at 400× magnification (D), ragged blue fibers following the SDH reaction at 200× magnification (E), no COX‐negative fibers following the sequential COX‐SDH reaction at 100× magnification (F). Transmission electron microscopy demonstrates marked mitochondrial accumulation, varied mitochondrial size and shape with abnormal cristae (G: 3,000× magnification, H: 10,000× magnification) (G) and paracrystalline (“parking lot”) mitochondrial inclusions (I, 40,000× magnification). D, Sequencing chromatograms portraying the homozygous c.1015G>A, p.(Ala339Thr) *COQ8A* variant identified in subject 1 (S1) that is heterozygous in her mother (M) and father (F) and absent in control DNA (C)

Subject 1 is a 6‐year‐old Caucasian girl, born to nonconsanguineous parents, who presented at birth with congenital hip dislocation. Hypotonia and developmental delay were noted at 14 months of age. She had achieved independent walking but was unstable by 3 years of age, with frequent falls and difficulty climbing stairs. No seizures were reported and familial pedigree analysis was unremarkable except for seven cases of glaucoma (Figure 1A); subject 1 has bilateral juvenile glaucoma and underwent trabeculectomy in her left eye. Her visual acuity is 20/60 (right eye) and 20/50 (left eye). Physical examination at 6 years of age revealed short stature (<3rd centile) and dysmorphic features, namely macrocephaly, dentinogenesis imperfecta, hypertelorism, slight exophthalmos, bluish sclera, and learning disability were reported. No ptosis or ophthalmoplegia were observed. Absent lower extremities deep tendon reflexes, hypotonia, and an abnormal wide‐based gait were noted. Electromyogram demonstrated myopathic motor unit potentials while nerve conduction studies were normal. Routine laboratory investigations revealed elevated serum creatine kinase: 1143 UI/L (normal <225 UI/L) while other investigations, including urinary organic acids and blood lactate were unremarkable.

Skeletal radiographs were consistent with delayed bone age, delayed posterior C1 and C2 fusion, and coxofemoral dislocation. Brain magnetic resonance imaging demonstrated decreased cerebral and cerebellar volume, supratentorial ventriculomegaly, and evidence of an incidental arachnoid cyst (Figure 1B). Computed tomography (CT) scan of the pelvis, thigh and legs demonstrated no muscle fat replacement. At 6 years of age, clinical suspicion of a mitochondrial disorder prompted commencement of therapeutic CoQ10 (100 mg/day) and deltoid biopsy for diagnostic investigations.

**Figure 2:**
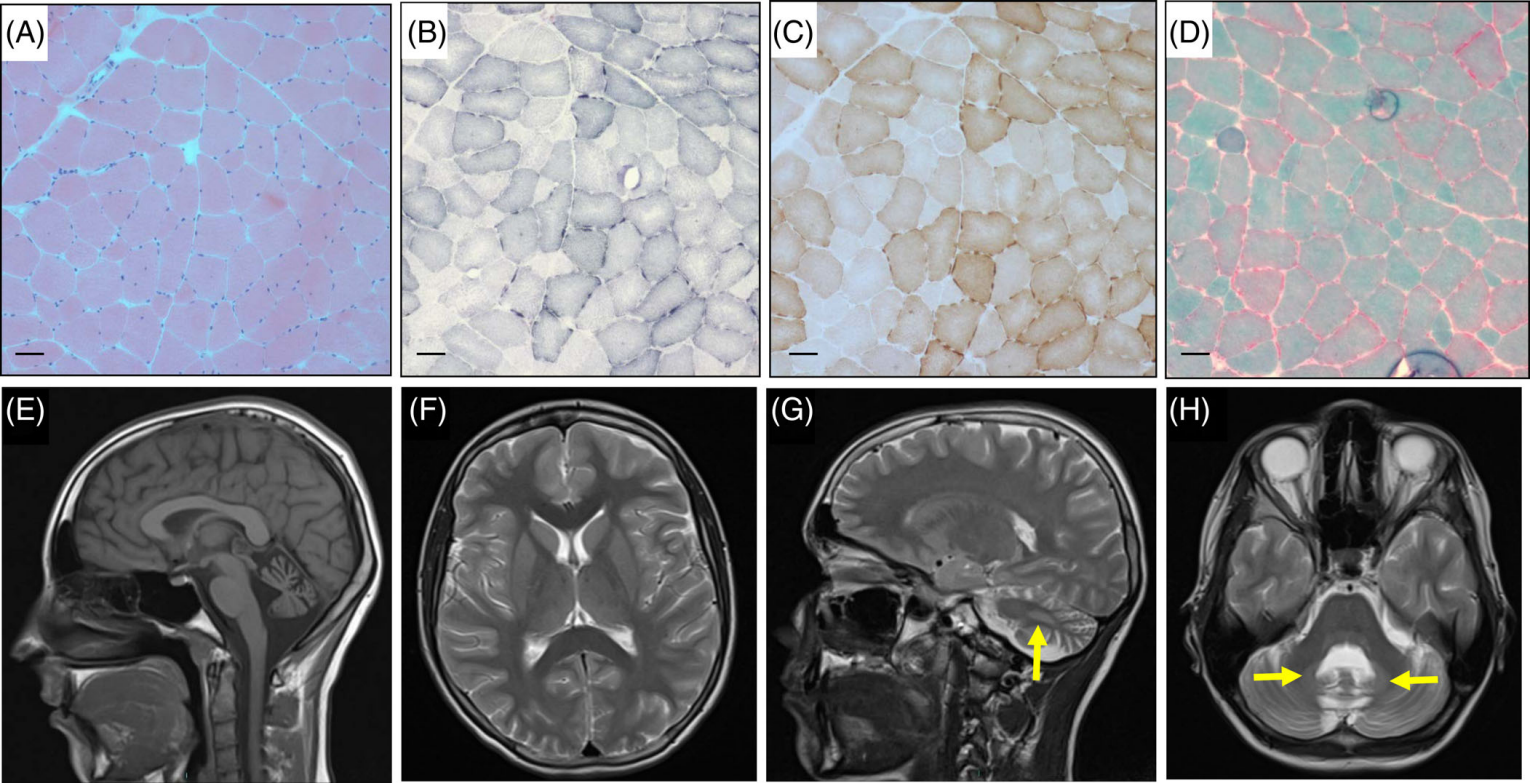
Muscle histochemistry and neuroimaging for subject 2. Histochemical analysis of muscle biopsy demonstrates normal architecture using H&E staining (panel A), fibers exhibiting signs of subsarcolemmal mitochondrial proliferation consistent with ragged red fiber pathology on the SDH reaction (panel B) and Gomori modified trichrome staining (panel D). Sequential COX‐SDH histochemistry demonstrated all fibers have preserved COX activity with ragged‐red pathology also evident (panel C); the scale bar on all images = 50 μm. Sagittal T1‐weighted MRI imaging shows marked cerebellar atrophy and widening of the fourth ventricle (panel E). T2‐axial view (panel F) shows normal cerebral cortices and no signal changes in basal ganglia. T2‐sagittal view (panel G) and T2‐axial view (panel H) show bilateral hyperintensities in the dentate nuclei (arrows)

Subject 2 is a 16‐year‐old white British female, who at 24 months of age was noted to have expressive speech difficulties and an unsteady gait with frequent falls. In early childhood, she was assessed by a community pediatric physician as having mild learning difficulties, a developmental coordination disorder and exercise intolerance and referred to occupational therapy. She had an intention tremor, difficulty tying shoe laces, poor tolerance of strenuous activity and although able to ride a bike she could only do so for short distances before fatiguing. She underwent cardiac assessment including an echocardiogram at 4 years of age which was normal. She was referred to neurology at 8 years of age. At that time, she complained of being tired after only walking short distances. She had an awkward gait and tended to stumble and trip much more often than children her age did; she was unable to skip or jump. Academically, subject 2 was 2‐3 years behind her peers for both writing and reading. On examination, she had a mild intention tremor and dysdiadochokinesis. She had generalized mild hypotonia. A brain MRI scan demonstrated pronounced cerebellar atrophy (Figure 2) though she remained stable for several years and was discharged back to community follow‐up at 13 years of age after a repeat MRI brain scan showed no progression. At 15 years of age, she was re‐referred to neurology following the onset of generalized tonic clonic seizures. The first episode was nocturnal and lasted ~10 minutes preceded by a period of incoherence. She had three further seizures over the following 3 months and a sleep EEG supported a possible left temporal onset and Levetiracetam was prescribed. She was found to be hypertensive (blood pressure: 199/100 [exercise], 135/85 [resting]) prompting treatment with antihypertensives and enalapril. Progressive exercise intolerance led to cardiac assessment at 15 years of age which revealed hypertension and mild left ventricular dysfunction (fractional shortening was 43%) with elevated lactate at 10.9 mmol/L (normal range < 2.0 mmol/L) not explained by the degree of cardiac dysfunction. She also started having generalized tonic‐clonic seizures and was commenced on anticonvulsant medication. Further metabolic investigations showed a persistently elevated lactate (7.31 mmol/L, normal: <2.0 mmol/L), elevated plasma alanine (790 μmol/L, normal range: 152–547 μmol/L), TCA metabolites in urine and normal CoQ10 in white cell lysates. The possibility of a mitochondrial etiology prompted muscle biopsy for characterization.

### 2.2 Histochemical and biochemical analyses of respiratory chain enzymes

Histochemical analysis of serial muscle biopsy sections was performed using standard protocols while biochemical analysis of respiratory chain enzyme activities in mitochondrial enriched muscle biopsy homogenates was performed as described previously.^10^ Assay of muscle CoQ10 levels was undertaken for subject 2 using methodology reported elsewhere.^11^

### 2.3 Molecular genetic analyses

Genetic analysis of the *COQ8A* gene (GenBank accession NM_020247.4) was undertaken using oligonucleotides targeting each coding exon (primer sequences supplied in Table S1, Tab A). Sanger sequencing was undertaken using the BigDye Terminator v3.1 kit (Life Technologies, Carlsbad, CA) and an ABI3130xl automated sequencer performed capillary electrophoresis according to the manufacturer's standard protocol (Life Technologies, Carlsbad, CA).

### 2.4 RNA extraction and cDNA analysis

RNA extraction and first strand synthesis was performed as reported previously^12^ using four overlapping primer pairs designed using Primer3 (Table S1, Tab B) and specific to the full length *COQ8A* transcript (GenBank accession NM_020247.4). Aberrant amplicons were subject to bandstab extraction, reamplification and resequencing. Visual inspection of the sequencing chromatograms was undertaken using FinchTV and BLAST was used to align the sequence to the human genome (GRCh38).

### 2.5 Long range PCR

Long range PCR was performed using the Promega GoTaq Long kit and 100 ng whole genomic DNA from Subject 2 and a healthy control to determine whether a deep intronic splicing variant was present. Primers were selected to amplify across the skipped exon 6 (exon 4F and exon 7R, Table S1, Tab A). Following confirmation of amplification using electrophoresis through a 1% agarose gel, Sanger sequencing was performed as per the manufacturers protocol to characterize the long‐range amplicon.

## 3 RESULTS

### 3.1 Histochemical and biochemical assessment of muscle biopsy

Histochemical analyses of the muscle biopsies from both subjects 1 and 2 report similar findings supportive of mitochondrial dysfunction. Both biopsies harbor a number of ragged‐red fibers (approximately 1% in subject 1 and 2% for subject 2), consistent with mitochondrial proliferation. Specifically, subject 1 muscle biopsy showed preserved general architecture with isolated atrophic fibers and focal necrosis (Figure 1C, Panels A and B), with small type 1 fibers and frequent lipid droplets (Figure 1C, Panel C). There was evidence of subsarcolemmal mitochondrial accumulation consistent with ragged‐red fiber pathology (Figure 1C, Panels D and E); no COX‐deficient fibers were observed (Figure 1C, Panel F). Muscle transmission electron microscopy demonstrated subsarcolemmal and intermyofibrillary mitochondrial accumulation with variation in size and shape; paracrystalline inclusions, dense granules, absent or abnormal cristae, lipid and glycogen deposits, myofiber splitting and areas of severe myofiber loss were also apparent (Figure 1C, Panels G‐I). Subject 2 muscle biopsy revealed normal architecture (Figure 2, Panel A) with approximately 2% of fibers demonstrating mitochondrial proliferation (Figure 2, Panels B‐D); again, no COX‐deficient fibers were observed (Figure 2, Panel C). Biochemical analysis of respiratory chain complex activities in muscle homogenates from subjects 1 and 2 also revealed striking similarities; both were found to have a marked combined complex II+III deficiency, calculated at 6% and 11% of mean control values, respectively. Citrate synthase activity was found to be elevated, consistent with mitochondrial proliferation. Determination of muscle CoQ10 levels was performed for subject 2 which confirmed a severe deficiency (5 pmol/mg, normal range 140‐580 pmol/mg).

### 3.2 Molecular genetic investigations

**Figure 3:**
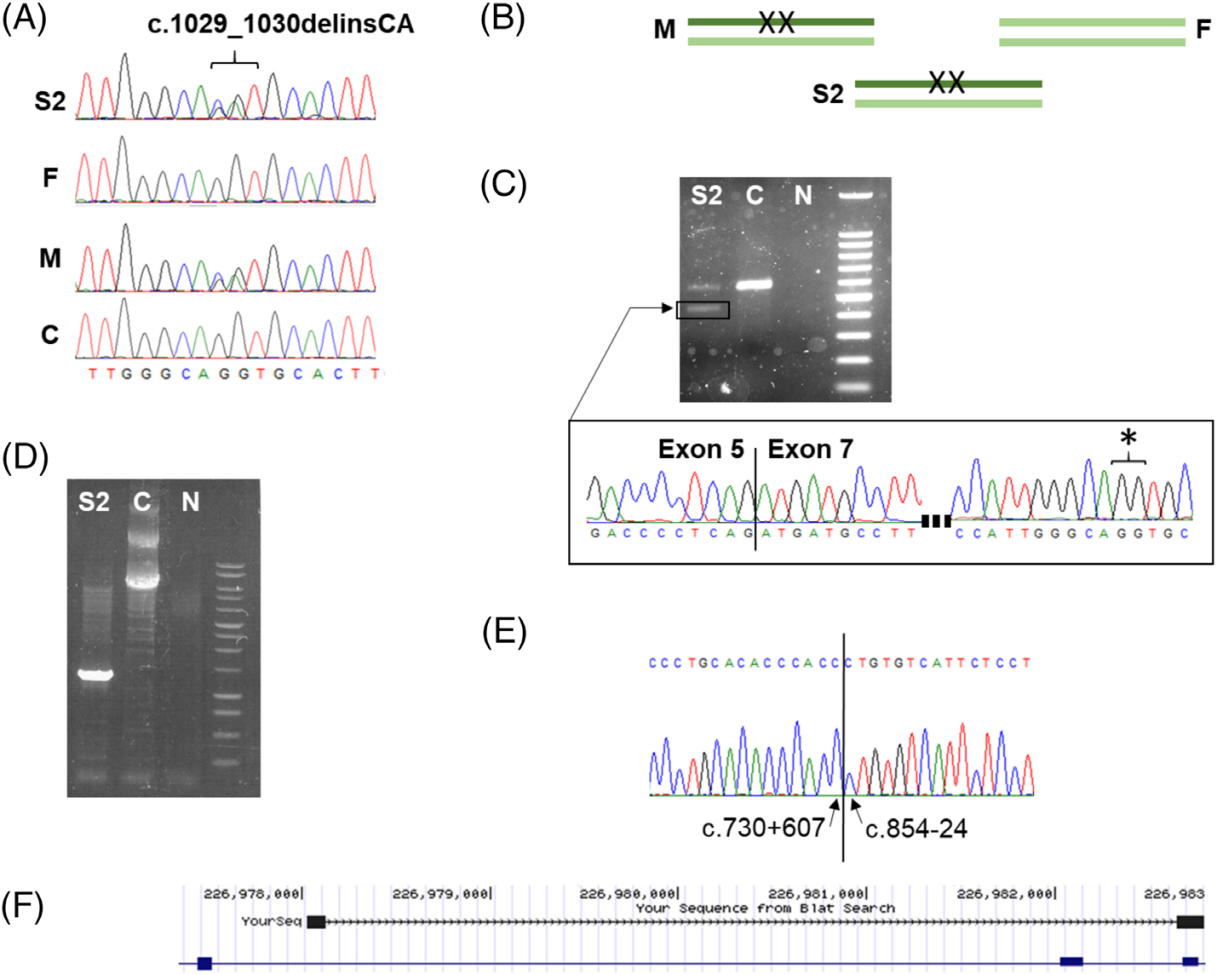
Molecular genetic findings for subject 2. A, Sanger sequencing of genomic DNA from subject 2 (S2), father (F), mother (M) and control (C) show maternal transmission of the c.1029_1030delinsCA dinucleotide variant that is absent in her father and healthy control. B, Schematic illustrating the maternally transmitted c.1029_1030delinsCA allele, where X indicates a sequence variant (sample abbreviations as before). C, Agarose electrophoresis of PCR amplified fibroblast‐derived cDNA demonstrates an aberrantly spliced product in subject 2 (S2) relative to an age and tissue matched control cDNA sample (C). N: no template control. DNA marker is 100 bp size standard (Promega cat G2101). Sanger sequencing of the aberrantly spliced amplicon confirms skipping of exon 6 (C, lower panel) and shows only wildtype sequence (GG) at nucleotides c.1029_1030 (denoted by an asterisk) confirming *in trans* variation. D, Agarose electrophoresis of genomic DNA amplified by long‐range PCR shows a smaller amplicon of size ~1.3 kb in the Subject (S2) relative to the control amplicon (C) with the anticipated size (5.7 kb), consistent with a genomic deletion. N: no template control. Promega 1 kb ladder was used as a size standard. E, Sanger sequencing of the ~1.3 kb amplicon from subject 2 confirmed a ~4.5 kb deletion, c.730+608_c.854‐25del, GRCh38: g.226978131_226982653del. F, BLAT search and IGV visualization of subject 2's deletion (dashed line) involving intron 5, exon 6 and intron 6

Sequencing analysis revealed a homozygous c.1015G>A, p.(Ala339Thr) *COQ8A* variant for subject 1; both parents were subsequently found to be carriers, thereby confirming autosomal recessive inheritance. Given that the c.1015G>A, p.(Ala339Thr) *COQ8A* variant is known to be pathogenic,^13^ no additional investigations were required to confirm the diagnosis. Sanger sequencing of subject 2's blood‐derived DNA sample demonstrated two heterozygous *COQ8A* variants involving adjacent nucleotides, c.1029G>C p.Gln343His and c.1030G>A p.Val344Met (Figure 3A). Although initially suspected to be biallelic, carrier testing of parental samples demonstrated that the variants occurred *in cis*, with maternal inheritance of a single heterozygous c.1029_1030delinsCA p.Gln343_Val344delinsHisMet variant (Figure 3B). Given that the clinical diagnosis was consistent with biallelic pathogenic *COQ8A* variants, additional experiments were performed to determine whether a second variant was present but not detectable using the Sanger sequencing strategy.

Initially, a fibroblast cell line was referred for cDNA investigations that revealed abnormal splicing, with skipping of exon 6 to create an in‐frame c.731_853del mRNA variant that is predicted to delete the p.Gly244_Gln284 residues from the protein (Figure 3C). Further interrogation of the sequence showed only wild type sequence at the c.1029_1030 locus (Figure 3D, denoted by an asterisk), thus establishing the aberrantly spliced allele to be *in trans* with the c.1029_1030delinsCA variant. A long‐range PCR was performed to permit screening of the intronic sequence for a putative splicing variant, however agarose electrophoresis revealed a large‐scale genomic deletion (Figure 3E). Sanger resequencing characterized the deletion break points as c.730+608 and c.854‐25del (GRCh38: g.226978131_226982653del) (Figure 3E), involving exon 6 and much of the intronic sequence of introns 5 and 6 (Figure 3F). Subject 2's father was found to be heterozygous for the c.730+608_854‐25del p.Gly244_Gln284del variant thereby confirming recessive inheritance of *COQ8A* variants in this family.

## 4 DISCUSSION

Ubiquinone, CoQ10, is a hydrophobic molecule composed from a reactive quinone head group and a lipid tail comprising 10 carbon isoprenoid repeats.^14^ It is present in all tissues, at varying levels and is vital for ATP production in tissues with high energetic demands, shuttling electrons from respiratory chain complexes I and II to complex III during oxidative phosphorylation.^15^ Primary CoQ10 deficiencies are caused by recessive pathogenic mutations in ubiquinone biosynthesis genes; to date pathogenic variants in nine genes are reported ‐ *PDSS1*,^16^ *PDSS2*,^17^ *COQ2*,^18^ *COQ4*,^19^ *COQ6*,^20^ *COQ7*,^21^ *COQ8A* (*ADCK3*),^22^ *COQ8B* (*ADCK4*),^23^ and *COQ9*.^24^ As with other classes of mitochondrial disease, age of onset is varied and there is considerable phenotypic heterogeneity associated with CoQ gene defects. Despite that, some genetic defects do correlate with characteristic symptoms, for example renal involvement is frequently observed in patients with biallelic *COQ2* variants^25^ while patients harboring recessive defects in *COQ8A* (*ADCK3*) often present with cerebellar ataxia.^16^ We report two unrelated female patients who presented in childhood. Subject 1 had subtle clinical signs of cerebellar ataxia with unsteadiness, frequent falls and a wide‐based gait, but no upper limb dysmetria, tremor or nystagmus. Subject 2 had more prominent features of cerebellar ataxia with similar gait abnormalities, but in addition, dysdiadochokinesis and intention tremor. Both subjects had pronounced complex II+III deficiencies, consistent with a clinical diagnosis of primary CoQ deficiency and additionally, subject 2 was found to have dramatically reduced CoQ10 levels in muscle. Both subjects were subsequently found to harbor recessive *COQ8A* defects and their symptoms were responsive to therapeutic CoQ10 which precipitated substantial clinical improvement in walking, gait stability and cognition. For subject 1, the initial therapeutic dosage of CoQ10 was 100 mg/day which precipitated pronounced clinical improvement—she was able to walk greater distances without falls. After 4 months of 100 mg/day CoQ10 therapy, the dosage was increased to 300 mg/day which demonstrated continuous improvement in gait. The therapeutic CoQ10 dosage for subject 2 was 1000 mg, three times daily, which improved energy levels, exercise tolerance and gait within a few months. Clinical improvements plateaued despite subsequent increases in CoQ10 dosage.

The homozygous c.1015G>A p.Ala339Thr *COQ8A* variant observed in subject 1 is reported once in the literature^13^ in a pediatric patient with ataxia, cognitive delay and CoQ10 deficiency; the patient harbored compound heterozygous *COQ8A* variants but neither variant interpretation nor a detailed description of the subject's clinical presentation was reported.^13^ The c.1015G>A p.Ala339Thr variant is absent on the Genome Aggregation Database (gnomAD, a repository of genetic variation from genomic sequencing projects) and the p.Ala339 residue is evolutionarily conserved to *C. elegans*, located in a highly conserved functional domain required for CoQ10 biosynthesis. Additionally, the c.1015G>A p.Ala339Thr variant is reported as “likely pathogenic” on ClinVar (SCV000262867); the variant satisfies criteria PM1, PM2, PP3, and PP4 according to the ACMG guidelines,^26^ fulfilling the requirements for a “likely pathogenic” status. The CADD scoring system was applied to the c.1015G>A p.Ala339Thr variant, revealing a score of 25.3 and therefore placing the p.Ala339Thr substitution within the top 0.5% of the most deleterious variants. While subject 1 was found to harbor a previously reported pathogenic homozygous *COQ8A* variant, the genetic diagnosis of subject 2 was considerably more challenging. The heterozygous c.1029G>C p.Gln343His and c.1030G>A p.Val344Met variants were established to be *in cis* and represented a single c.1029_1030delinsCA p.Gln343_Val344delinsHisMet variant, highlighting the importance of establishing phase. Initial investigations revealed that the c.1029G>C and c.1030G>A variants are very rare on the gnomAD database (n = 1 and n = 4 heterozygous cases, respectively); visual inspection of the raw sequencing data pertaining to the gnomAD hits revealed the c.1029G>C and c.1030G>A variants were *in cis* in one individual and the c.1030G>A variant occurs as an isolated variant in two cases, which suggests that the c.1029G>C variant occurred as an ancestral mutation on an allele already harboring a c.1030G>A variant. The Gln343 and Val344 residues are both conserved to *S. cerevisiae*, highlighting their functional importance, reinforced by additional *in silico* evidence. Classification of the two variants according to the ACMG guidelines^26^ does not obviously preclude the functional importance of either amino acid (Figure S1), and it is not possible to know whether an allele harboring both the p.Gln343His and p.Val344Met variants is associated with a more severe phenotype versus either variant in isolation. Although the c.1029G>C p.Gln343His, c.1030G>A p.Val344Met and c.1029_1030delinsCA p.Gln343_Val344delinsHisMet variants are absent on ClinVar, a c.1028A>G p.Gln343Arg substitution is reported as “likely pathogenic” on ClinVar (RCV000195825.1).

Although cDNA investigations revealed skipping of exon 6, the causative defect was not a consequence of disrupted splice motifs per se, but a large‐scale intragenic deletion, itself representing a novel mechanism of *COQ8A* pathology. The large‐scale 4.5Kb deletion is predicted to cause an in‐frame deletion of residues Gly244‐Gln284 in the COQ8A protein. 25/42 residues (60.0%) in exon 6 are conserved to *C. elegans* and 15/42 residues (35.7%) are conserved to *S. cerevisiae*, supporting the functional importance of this locus. The g.226978131_226982653del is unreported on gnomAD,^27^ ClinVar,^28^ or the Database of Genomic Variants (DGV).^29^ The c.730+608_854‐25del, c.1015G>A, and c.1029_1030delinsCA *COQ8A* variants have been submitted to ClinVar (Accession numbers: SCV000995085, SCV000995086 and SCV000995087).

The presence of biallelic *COQ8A* variants confirms the clinical diagnosis of a primary CoQ10 deficiency for both individuals. In addition to cerebellar ataxia, subject 1 presented with a number of additional features including hip dysplasia, macrocephaly, dentinogenesis imperfecta, hypertelorism, and exophthalmos; these dysmorphic features are not consistent with previous *COQ8A*‐deficient phenotypes. While they may be associated with defective *COQ8A*, it is more probable that they are the consequence of a second gene defect or genomic copy number variation but additional investigations have not yet been undertaken. The identification of a very rare variant occurring in the homozygous state in an extensive pedigree with no obvious consanguinity is unusual although not unprecedented^30^; we postulate that the c.1015G>A c.Ala339Thr *COQ8A* variant may represent a founder mutation given that carrier testing of her unrelated parents supports recessive inheritance. Subject 2 presented with characteristic features of recessive *COQ8A* defects, namely cerebellar ataxia and mild cognitive impairment, but identifying the causative genetic defect was challenging. Despite initially identifying two heterozygous variants, both were *in cis* and therefore were not sufficient to confirm a genetic diagnosis. cDNA studies confirmed aberrant splicing, attributable not to defects involving splicing enhancer or silencer motifs, but an intragenic deletion. To the best of our knowledge, this is the first case of an intragenic genomic deletion in the *COQ8A* gene. As diagnostic testing moves towards genomics as a first line of investigation, this case highlights the importance of carrier testing and the requirement for additional analyses to establish the correct genetic diagnosis. Many NGS sequencing strategies involve short reads and, using such an approach, it is likely that the intragenic deletion in subject 2 would have evaded detection. Biochemical evaluation of CoQ10 and mitochondrial respiratory chain activities using muscle biopsy precipitated the pursuit of *COQ8A* variants, demonstrating the utility of muscle biopsy despite its increasing displacement as a frontline test. As with other subjects with reported primary CoQ10 deficiency, therapeutic administration of CoQ10 has ameliorated specific symptoms in both patients. This underlines the importance of rapid identification of the correct genetic diagnosis as similar patients are likely to respond well to CoQ10 therapy.

## CONFLICT OF INTEREST

The authors have no conflicts of interest to disclose. All authors have read and approved the submitted manuscript.

## AUTHOR CONTRIBUTIONS

Conception and research design: Ana Cotta, Charlotte Alston, and Robert Taylor. Data collection: Ana Cotta, Charlotte Alston, Sidney Baptista‐Junior, Julia Paim, Elmano Carvalho, Monica Navarro, Marie Appleton, Jaquelin Valicek, Antonio da‐Cunha‐Junior, Maria Lima, Alessandra de la Rocque Ferreira, Reinaldo Takata, Iain Hargreaves and Germaine Pierre. Contribution of data and analysis: Ana Cotta, Charlotte Alston, Sidney Baptista‐Junior, Julia Paim, Elmano Carvalho, Monica Navarro, Marie Appleton, Iain Hargreaves, Yi Shiau Ng, Jaquelin Valicek, Antonio da‐Cunha‐Junior, Maria Lima, Alessandra de la Rocque Ferreira, Reinaldo Takata, and Gráinne Gorman. Performed the analysis: Ana Cotta, Charlotte Alston, Sidney Baptista‐Junior and Marie Appleton. Manuscript preparation: Ana Cotta, Charlotte Alston, Robert McFarland, Germaine Pierre, Gráinne Gorman and Robert Taylor.

## ETHICAL APPROVAL STATEMENT

All procedures followed were in accordance with the ethical standards of the responsible committee on human experimentation (institutional and national) and with the Helsinki Declaration of 1975, as revised in 2000.

## PATIENT CONSENT STATEMENT

Informed consent was obtained from all patients for being included in the study.

## Supporting information


**Table S1** Oligonucleotides used for identification of COQ8A (NM_020247.4) variants in Subjects 1 and 2.

**Figure S1** Classification of the c.1029G>C and c.1030G>A COQ8A variants identified in Subject 2 according to ACGS guidelines.

**Figure S2** Supplementary Information.
