# Supplementary material for: Early‐onset coenzyme Q10 deficiency associated with ataxia and respiratory chain dysfunction due to novel pathogenic COQ8A variants, including a large intragenic deletion
Source: JIMD Rep. 2020 Jun 2;54(1):45–53. doi: 10.1002/jmd2.12107 (PMC7358671; doi:10.1002/jmd2.12107)
Supplement: Supplementary file 2 — Figure S1 Classification of the c.1029G>C and c.1030G>A COQ8A variants identified in Subject 2 according to ACGS guidelines. [file JMD2-54-45-s002.docx]

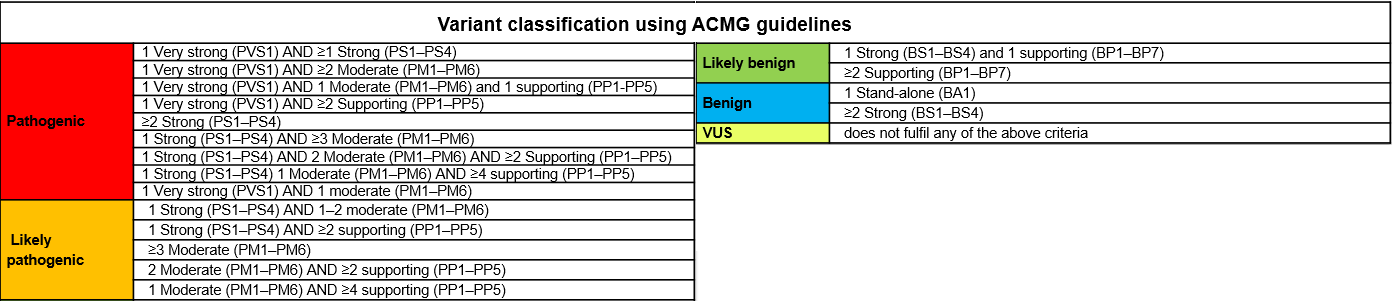

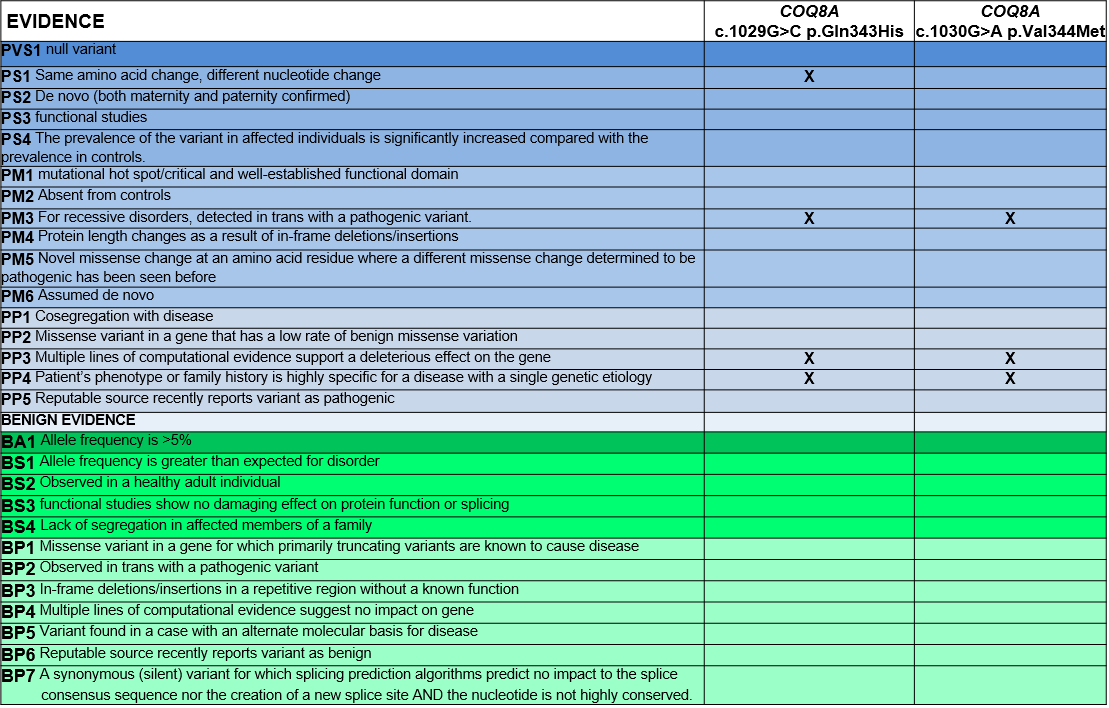


**Supplemental Figure S1.** Classification of the c.1029G>C and c.1030G>A COQ8A variants identified in Subject 2 according to ACGS guidelines.
