# Supplementary figures and images for: Early‐onset coenzyme Q10 deficiency associated with ataxia and respiratory chain dysfunction due to novel pathogenic COQ8A variants, including a large intragenic deletion
Source: JIMD Rep. 2020 Jun 2;54(1):45–53. doi: 10.1002/jmd2.12107 (PMC7358671; doi:10.1002/jmd2.12107)

## Slide 1
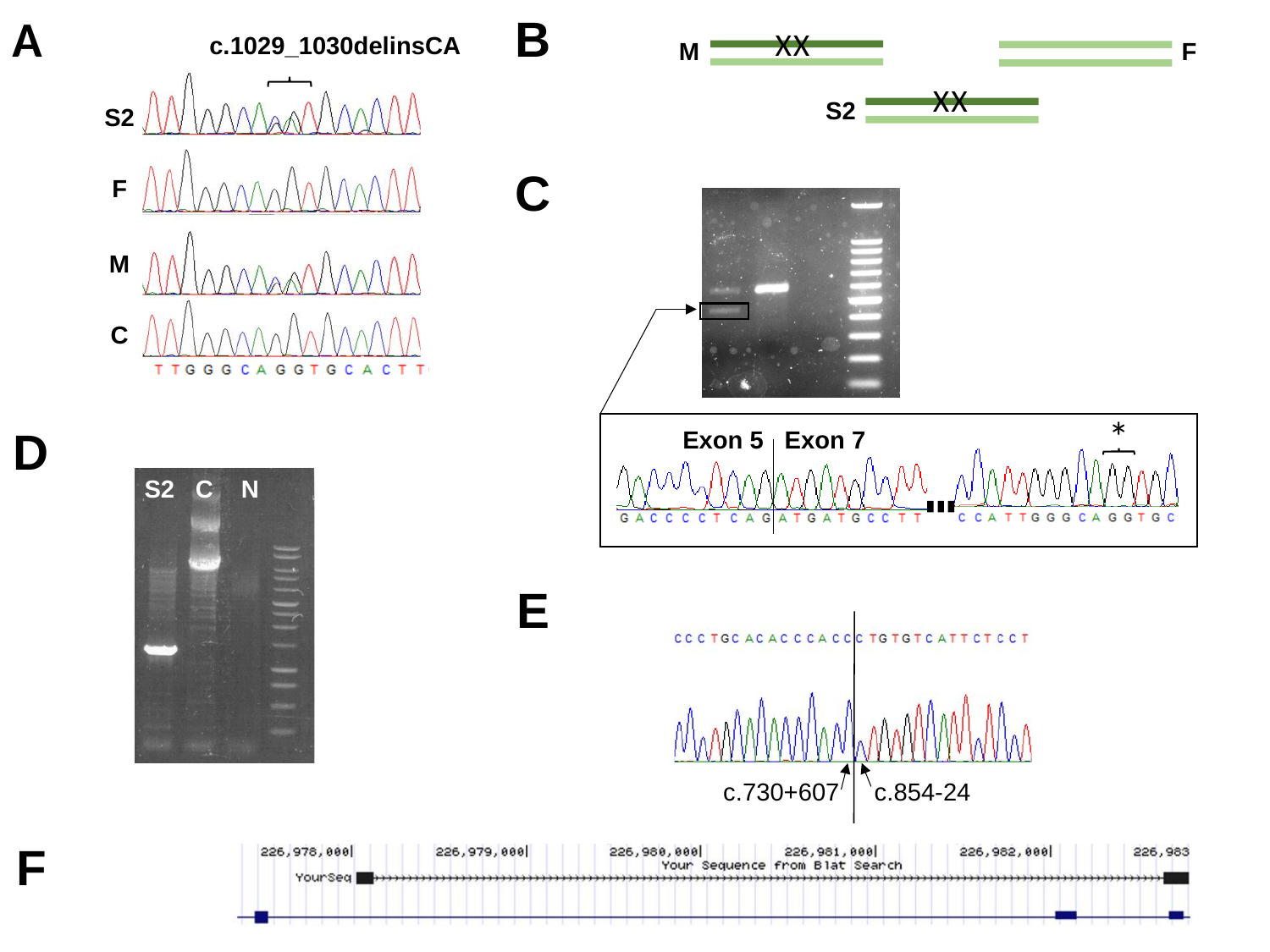

A
B
X
X
X
X
c.1029_1030delinsCA
M
F
S2
S2
C
F
S2 C N
M
C
*
D
Exon 5 Exon 7
S2 C N
E
c.730+607
c.854-24
F

Supplement: Supplementary file 3 — Figure S2 Supplementary Information. [file JMD2-54-45-s003.pptx]
